# Editorial: Artificial Intelligence in Positron Emission Tomography

**DOI:** 10.3389/fmed.2022.848336

**Published:** 2022-01-31

**Authors:** Hanyi Fang, Kuangyu Shi, Xiuying Wang, Chuantao Zuo, Xiaoli Lan

**Affiliations:** ^1^Department of Nuclear Medicine, Union Hospital, Tongji Medical College, Huazhong University of Science and Technology, Wuhan, China; ^2^Hubei Province Key Laboratory of Molecular Imaging, Wuhan, China; ^3^Department of Nuclear Medicine, University of Bern, Bern, Switzerland; ^4^Department of Informatics, Technical University of Munich, Munich, Germany; ^5^School of Computer Science, The University of Sydney, Sydney, NSW, Australia; ^6^PET Center and National Clinical Research Center for Aging and Medicine, Huashan Hospital, Fudan University, Shanghai, China

**Keywords:** artificial intelligence, molecular imaging, positron emission tomography, oncology, neurology

Smartphones, smart homes, and intelligent navigation are all examples of important applications of artificial intelligence (AI) in our daily life. AI was initially introduced in the 1950s, with the development of understanding and redefinition. AI is currently defined as a new technological science that studies and develops theorems, methods, technologies, and application systems that are used to simulate, extend, and enhance human intelligence (1).

We have witnessed the rapid advancement of AI, and its research and application in medical care, especially processing and analysizing the medical images is in the ascendant. In comparison to computed tomography (CT) and magnetic resonance imaging (MRI), which are more accessible and easier to standardize the acquisition processes, the positron emission tomography (PET) is more expensive and less broadly accessible, and its more complicated technical operation process poses difficulty on standardizing the image acquisition. Though the research and application of AI in PET is relatively slower, since PET is such an essential field of molecular imaging, AI in PET imaging is attracting substantial research attention and becoming a research hotspot. At the level of technology, image post-processing, including image standardization, normalization, wavelet transformation, Gaussian transformation, and feature preprocessing, have been studied with aims to solve the challenges posed by the parameter and quality variations and differences when imaging with PET scanners from different manufacturers, instrument models, and imaging technologies. The AI-empowered segmentation techniques have further improved the stability of AI features and the repeatability of AI researches (2, 3). To address the needs of clinical applications, by mining deeply into image features, combining population and clinical evidence, and constructing machine learning models, AI in PET has been developed for lesion detection and boundary delineation, diagnosis and differential diagnosis, risk prediction and prognostic evaluation, and even the prediction of clinical gene or molecular typing (1, 4–7).

This Research Topic comprises 11 publications that emphasized how AI supports PET image processing and analysis. Recently, numerous research groups have been focusing on the use of AI in PET image interpretation, such as lesion detection. Kawakami et al. applied an object deep learning (DL) detection model, You Only Look Once Version 2 (YOLOv2), to detect the physiological and abnormal uptake in ^18^F-FDG PET. Results showed that the physiological uptake on MIP images was recognized quickly and precisely (Kawakami et al.). The abnormal uptake detected by YOLOv2 was with a high coverage rate of that manually identified (Kawakami et al.). The precise detection and fast response would be a useful tool in disease diagnosis. The maximal standardized uptake value (SUVmax) is the most commonly used parameter to interpret images and evaluate lesions in the daily diagnostic reports. In a preliminary study, Hirata et al. sought to define a precise SUVmax as an identifier to locate lesions. Although it is difficult to identify the lesions when the SUVmax < 2 in ^18^F-FDG PET scans, this approach could help for the construction of the AI training dataset (Hirata et al.).

In oncology, AI has been used for diagnosis, differential diagnosis, and cancer staging. Satoh et al. used texture analysis in a retrospective study to compare the diagnosis ability of breast cancer between the high-resolution dedicated breast PET (dbPET) and whole-body PET/CT. They demonstrated that both PET-based texture analysis of dbPET and whole-body PET/CT had comparable classification power for the diagnosis of breast cancer (Satoh et al.). Fan et al. evaluated the value of texture analysis in the differential diagnosis of spinal metastases of ^18^F-FDG PET/CT, and indicated that the combination of machine learning and texture parameters was more accurate than manual diagnosis. Zheng et al. applied a radiomics model in ^18^F-FDG PET/CT to predict pathological mediastinal lymph node (pN) staging in patients with non-small cell lung cancer (NSCLC), and demonstrated an encouraging conclusion, suggesting that the pN staging and prediction have the potential to help with therapeutic planning. Apart from PET/CT, another nuclear medicine imaging method—single-photon emission computed tomography/computed tomography (SPECT/CT) also plays a role in the differentiation of benign and malignant tumors. Jin et al. investigated the feasibility of SPECT/CT images based on radiomics in differentiating bone metastases from benign bone lesions in patients with tumors. Both SPECT and SPECT/CT models showed better diagnostic accuracy than manual classification in the training and validation groups of patients diagnosed with vertebral bone metastases or benign bone lesions, indicating a new way in disease staging and treatment planning (Jin et al.).

In neurology, AI has also been applied to help for the distinguishment and mechanism research of neurodegenerative diseases. Xie et al. revealed a pattern of changes in β-amyloid (Aβ) deposition in cognitively normal healthy aging, which could be used to distinguish physiological changes from pathophysiological changes and help investigate the mechanism of Alzheimer's disease (AD). Zhou et al. presented a new deep learning model based on rate-distortion theory and an extreme learning machine model to distinguish AD, mild cognitive impairment (MCI), and normal controls (NC) in ^18^F-AV45 PET/MR. This new deep learning model achieved higher accuracy, sensitivity, specificity, and area under curve (AUC) to separate AD, MCI, and NC groups than the previous models assessed (Zhou et al.).

The applications of AI are more than image processing, analysis, and interpretation, and also embrace searching for electronic health records, laboratory tests, and other information related to patients, which could assist physicians make optimal and personalized medical decisions for patients. With such abilities, AI provides more opportunities for assessing therapeutic responses and predicting survival rates. Tang et al. investigated the metabolic profiles of extratemporal in drug-resistant temporal lobe epilepsy (TLE) and efficiently predicted the surgery failure of TLE patients through PET, which could be used as predictive models for epilepsy surgery. Pinochet et al. evaluated the performance of a research prototype called PET Assisted Reporting System (PARS), which is based on a convolutional neural network, in clinical research. The PARS, determined total tumor metabolic volumes (TMTVs) on ^18^F-FDG PET, was predictive of prognosis in patients with diffuse large B-cell lymphoma (DLBCL), but the evaluation efficacy was still needed to be enhanced and validated in miscellaneous cancers (Pinochet et al.). Yang et al. developed a radiomics score using the least absolute shrinkage and selection operator (LASSO) regression analysis in ^18^F-FDG PET/CT-derived radiomic features, which proved to be a useful tool for predicting overall survival (OS) in adult hemophagocytic lymphohistiocytosis (HLH). Combining the radiomics score with the clinical parameters even performed better for predicting 6-month survival (Yang et al.). Apart from the above studies, AI can also assist to evaluate therapeutic responses and predict prognosis in other emerging treatments, including immunotherapy (8) and peptide radio receptor therapy (PRRT) (9).

Furthermore, with the powerful searching ability, AI can optimize the workflow and provide more detailed and organized information of patients, making it convenient for doctors to assess patient status more efficiently and precisely. Improtantly, AI plays a supportive role to relieve the physicians from labor-intensive but less cognitively demanding routine tasks, allowing them to focus on more mental work, such as patient care and image interpretation (1). Another step that is inseparable from AI in PET is imaging data processing, especially when it comes to standardizing imaging acquisition and reconstruction procedures. The repeatability of AI analysis in multicenter settings is essential to clinical translation. Zwanenburg performed a meta-analysis to evaluate the repeatability of PET imaging biomarkers (10). Based on the results, variations in the image acquisition, reconstruction, segmentation, and processing strongly affect the reliability of image biomarkers in models of different PET centers (10).

Although AI has tremendous potential in PET, it is critically important to be aware of its limitations. First, the reproducibility and reliability of AI algorithms are required. With the modeling becoming more and more complicated, the “black box” nature of AI makes it difficult to understand and explain the results of many AI models, especially in some DL models (11). The current trustworthy AI for health care prefers the explainability and stability of diverse and unknown data (12). Second, AI needs massive annotated data for learning and growing, which makes AI less reliable in small datasets. Currently, standard datasets, such as the Alzheimer's Disease Neuroimaging Initiative (ADNI) that is available to the public, are still relatively rare and in high demands. More open standard datasets will promote the development of AI. Last but not the least, there are still many ethical issues that need to be discussed, such as who is responsible for the legal and ethical issues if the AI diagnosis turns out to be wrong?

Despite the above limitations, it is obvious that AI plays a unique role in every step of PET, including patient information management, drug synthesis and administration, image acquisition and processing, as well as report interpretation. AI can not only be used for helping make clinical decisions, but also as a research helper for discovering and investigating novel molecular biomarkers and their mechanisms in various diseases (13). With the powerful impact of AI in medical imaging, many physicians, especially radiologists, are concerned to be replaced by AI in the future. In fact, as Nensa et al. said, we should perceive the change as an opportunity rather than a threat (1). In the near future, or even at the moment, medical imaging physicians should not only simply focus on describing what images showing, but also need to pay more attention to all available information and data of patients, and conduct comprehensive analysis and interpretation to diseases diagnosis and therapeutic efficacy assessment or prediction, which greatly assist in providing more precise and personalized medical care for every individual patient.

## Author Contributions

HF wrote the manuscript. XL, KS, XW, and CZ edited the manuscript. All authors have made a substantial, direct, and intellectual contribution to the work and approved it for publication.

## Funding

This work was supported by the Opening Foundation of Hubei Key Laboratory of Molecular Imaging (2020fzyx003).

## Conflict of Interest

The authors declare that the research was conducted in the absence of any commercial or financial relationships that could be construed as a potential conflict of interest.

## Publisher's Note

All claims expressed in this article are solely those of the authors and do not necessarily represent those of their affiliated organizations, or those of the publisher, the editors and the reviewers. Any product that may be evaluated in this article, or claim that may be made by its manufacturer, is not guaranteed or endorsed by the publisher.
